# Supplementary material for: A neurocomputational account of the link between social perception and social action
Source: eLife. 2025 Apr 16;12:RP92539. doi: 10.7554/eLife.92539 (PMC12002797; doi:10.7554/eLife.92539)
Supplement: Supplementary file 1. [file elife-92539-supp1.docx]

**Supplementary file 1.** Estimates of the computational model of social perception (hyper-mean parameter estimates, means of the posterior distributions with 95% Highest Density Interval, HDI).

|  | **Condition** | | |
| --- | --- | --- | --- |
| **Parameter** | **Merit** | **Need** | **Control** |
| *bias* | 0.29  [0.10, 0.47] | -0.12  [-0.31, 0.06] | 0.09  [-0.05, 0.23] |
| *S_merit_* | **2.94**  [2.48, 3.35] | 0.39  [-0.07, 0.85] | 0.22  [0.11, 0.33] |
| *S_need_* | 0.39  [-0.002, 0.78] | **3.31**  [2.93, 3.64] | -0.12  [-0.22, -0.02] |
| *S_control_* | 0.42  [0.21, 0.64] | 0.26  [0.11, 0.41] | **4.57**  [4.22, 4.78] |
| *z* | 0.53  [0.51, 0.54] | 0.60  [0.59, 0.62] | 0.55  [0.53, 0.56] |
| *a* | 1.83  [1.75, 1.92] | 1.85  [1.77, 1.93] | 1.76  [1.68, 1.84] |
| *ndt* | 0.59  [0.52, 0.66] | 0.62  [0.55, 0.69] | 0.60  [0.54, 0.67] |

*Note. bias* = overall perceptual bias; *S_merit_* = merit sensitivity, *S_need_* = need sensitivity, *S_control_* = control sensitivity), bold indicates task-relevant sensitivity scores, *z* = starting bias, *a* = difference between barriers, *ndt* = non-decision time
