# Supplementary material for: A neurocomputational account of the link between social perception and social action
Source: eLife. 2025 Apr 16;12:RP92539. doi: 10.7554/eLife.92539 (PMC12002797; doi:10.7554/eLife.92539)
Supplement: Supplementary file 2. [file elife-92539-supp2.docx]

**Supplementary file 2.** Whole-brain searchlight decoding of need, merit, and control inferences (social perception task).

|  | **Brain Region** | **Side** | **T** | **k** | **MNI (peak)** | | |
| --- | --- | --- | --- | --- | --- | --- | --- |
|  |  |  |  |  | **x** | **y** | **z** |
| [Merit vs. Control] |  |  |  |  |  |  |  |
|  | TPJ / angular gyrus | L | 6.08 | 3885 | -52 | -64 | 44 |
|  | TPJ / angular gyrus | R | 5.48 | 2241 | 48 | -60 | 32 |
|  | DMPFC | L | 5.37 | 1129 | -6 | 68 | 12 |
|  | DMPFC | R | 4.94 | 1265 | 14 | 42 | 40 |
|  | Superior frontal sulcus | R | 5.05 | 678 | 26 | 12 | 60 |
| [Need vs. Control] |  |  |  |  |  |  |  |
|  | Occipito-temporal cortex | L/R | 10.94 | 129.326 | -56 | -60 | -10 |
|  | *Note: the cluster includes the bilateral TPJ, DMPFC, temporal poles and posterior cingulate cortex |  |  |  |  |  |  |
|  |  |  |  |  |  |  |  |
| [Merit vs. Need] |  |  |  |  |  |  |  |
|  | - | - | - | - | - | - | - |

*Note.* Results are reported at a statistical threshold of p < 0.001 at the voxel level, FWE corrected at the cluster level at p < 0.05. No brain region allowed decoding Merit vs. Need judgments at this threshold. L = Left hemisphere, R = Right hemisphere, MNI = Montreal Neurological Institute, k = cluster size in voxels. Only peak coordinates of clusters are reported. Sample size n = 44, TPJ = temporoparietal junction, DMPFC = dorsomedial prefrontal cortex
