# Supplementary material for: A neurocomputational account of the link between social perception and social action
Source: eLife. 2025 Apr 16;12:RP92539. doi: 10.7554/eLife.92539 (PMC12002797; doi:10.7554/eLife.92539)
Supplement: Supplementary file 3. [file elife-92539-supp3.docx]

**Supplementary file 3.** Brain regions activated during merit perceptions (social perception task) that reflect individual differences in merit sensitivity (*S_merit_*) estimated in the computational model of social perception (in the n = 25 participants with overlapping altruistic choice task data).

|  | **Brain Region** | **Side** | **T** | **k** | **MNI (peak)** | | |
| --- | --- | --- | --- | --- | --- | --- | --- |
|  |  |  |  |  | **x** | **y** | **z** |
| ***Covariate: Merit Sensitivity*** |  |  |  |  |  |  |  |
| [Merit > Control] | Temporoparietal junction (TPJ) | R | 4.48 | 157 | 48 | -62 | 22 |
|  | Precuneus | R | 5.66 | 380 | 6 | -60 | 56 |
|  | Frontal eye fields | R | 5.12 | 342 | 22 | 0 | 54 |
|  | Premotor cortex | R | 5.84 | 113 | 34 | -46 | 58 |
| [Need > Control] | - | - | - | - | - | - | - |
| [Merit > Need] | - | - | - | - | - | - | - |

*Note.* Cluster peaks are reported at a statistical threshold of p < 0.001 at the voxel level, p < 0.05 FWE corrected at the cluster level. L=Left hemisphere, R=Right hemisphere, MNI=Montreal Neurological Institute, k=cluster size in voxels.
