# Supplementary material for: A neurocomputational account of the link between social perception and social action
Source: eLife. 2025 Apr 16;12:RP92539. doi: 10.7554/eLife.92539 (PMC12002797; doi:10.7554/eLife.92539)
Supplement: Supplementary file 4. [file elife-92539-supp4.docx]

**Supplementary file 4.** Logistic mixed models predicting generous choice (0/1).

|  | **Model 1**  **(Null Model)** | | **Model 2**  **(Winning Model)** | | **Model 3**  **(Interaction Model)** | |
| --- | --- | --- | --- | --- | --- | --- |
|  | Estimate | S.E. | Estimate | S.E. | Estimate | S.E. |
| (Intercept) | **-0.81*****  [-1.25, -0.37] | 0.23 | **-1.55*****  [-2.01, -1.08] | 0.24 | **-1.48*****  [-1.95, -1.00] | 0.24 |
| need [high] | - | - | **0.53*****  [0.43, 0.63] | 0.05 | **0.40*****  [0.21, 0.58] | 0.10 |
| merit [unknown] | - | - | **0.63*****  [0.50, 0.75] | 0.07 | **0.52*****  [0.33, 0.71] | 0.10 |
| merit [high] | - | - | **0.71*****  [0.58, 0.83] | 0.07 | **0.60*****  [0.42, 0.79] | 0.10 |
| need [high] × merit [unknown] | - | - | - | - | 0.19  [-0.06, 0.45] | 0.13 |
| need [high] × merit [high] | - | - | - | - | 0.19  [-0.07, 0.45] | 0.13 |
| self | - | - | - | - | - | - |
| other | - | - | - | - | - | - |
| fairness | - | - | - | - | - | - |
| R^2^ | 0 |  | 0.33 |  | 0.33 |  |
| AIC | 8973 |  | 8742 |  | 8743 |  |
| BIC | 8987 |  | 8777 |  | 8792 |  |

*Note.* Significant effects indicated in bold. * p < 0.05 ** p < 0.01 *** p < 0.001. Sample size n = 28. The model’s intercept corresponds to need = low, merit = low, self = 0, other = 0 and fairness = 0. 95% Confidence Intervals (displayed in brackets) and p-values were computed using a Wald z-distribution approximation.
