# Supplementary material for: A neurocomputational account of the link between social perception and social action
Source: eLife. 2025 Apr 16;12:RP92539. doi: 10.7554/eLife.92539 (PMC12002797; doi:10.7554/eLife.92539)
Supplement: Supplementary file 5. [file elife-92539-supp5.docx]

**Supplementary file 5**. Model-estimated weights of choice-relevant attributes (*w_self_*, *w_other_*, *w_fairness_*) and drift intercept bias (*w_0_*) in the altruism task at the participant level (computational model of altruistic choice, n = 28).

| **Attributes** | **Estimates** | **Mean** | **SD** |
| --- | --- | --- | --- |
| *w*_0_ | Baseline | 0.34 | 0.44 |
|  | Δ_Need_ | -0.03 | 0.06 |
|  | Δ_High_ *_Merit_* | -0.09 | 0.02 |
|  | Δ_Low_ *_Merit_* | -0.12 | 0.04 |
| *w*_self_ | Baseline | 0.96 | 0.72 |
|  | Δ_Need_ | -0.08 | 0.13 |
|  | Δ_High_ *_Merit_* | -0.04 | 0.07 |
|  | Δ_Low_ *_Merit_* | 0.12 | 0.13 |
| *w*_other_ | Baseline | 0.31 | 0.52 |
|  | Δ_Need_ | 0.07 | 0.10 |
|  | Δ_High_ *_Merit_* | 0.03 | 0.03 |
|  | Δ_Low_ *_Merit_* | -0.28 | 0.48 |
| *w*_fairness_ | Baseline | 0.35 | 0.37 |
|  | Δ_Need_ | -0.02 | 0.03 |
|  | Δ_High_ *_Merit_* | -0.03 | 0.03 |
|  | Δ_Low_ *_Merit_* | -0.12 | 0.10 |

*Note*. For hyper-mean parameter estimates of the computational model of altruistic choice (means of the posterior distributions with 95% Highest Density Interval, HDI), see Supplementary file 6. To reconstruct Figure 5B, take [Baseline + Δ_Low Merit_], [Baseline], and [Baseline + Δ_High Merit_]. To reconstruct Figure 5C, take [Baseline – Δ_Need_], and [Baseline + Δ_Need_].
