# Supplementary material for: A neurocomputational account of the link between social perception and social action
Source: eLife. 2025 Apr 16;12:RP92539. doi: 10.7554/eLife.92539 (PMC12002797; doi:10.7554/eLife.92539)
Supplement: Supplementary file 6. [file elife-92539-supp6.docx]

**Supplementary file 6.** Hyper-mean parameter estimates (computational model of altruistic choice).

| **Parameter** | **Fixed** | **Low Need** | **High Need** | **Low Merit** | **High Merit** |
| --- | --- | --- | --- | --- | --- |
| *w*_0_ | – | 0.38  [0.16, 0.59] | 0.31  [0.09, 0.52] | -0.12  [-0.19, -0.06] | -0.10  [-0.14, -0.05] |
| *w_self_* | – | 1.05  [0.74, 1.35] | 0.89  [0.58, 1.20] | 0.12  [0.02, 0.22] | -0.04  [-0.11, 0.03] |
| *w_other_* | – | 0.24  [0.02, 0.47] | 0.38  [0.16, 0.61] | -0.28  [-0.49, -0.07] | 0.03  [-0.01, 0.08] |
| *w_fairness_* | – | 0.37  [0.21, 0.53] | 0.33  [0.17, 0.49] | -0.12  [-0.18, -0.06] | -0.03  [-0.06, 0.00] |
| *z* | 0.50  [0.48, 0.53] | – | – | – | – |
| *a* | 2.61  [2.43, 2.80] | – | – | – | – |
| *ndt* | 0.61  [0.53, 0.69] | – | – | – | – |

*Note.* Means of the posterior distributions with 95% HDI in brackets. *w* = drift rates (*w*_0_ = bias, *w_self_* = self outcome, *w_other_* = partner outcome, *w_fairness_* = fairness between self and partner outcome), *z* = starting bias, *a* = difference between barriers, *ndt* = non-decision time. To capture how attribute weights (w_self_, w_other_, w_fairness_) differed across conditions, our computational model estimated four separate drift parameters for each attribute: 1) baseline sensitivity for the unknown partner; 2) an additive term related to the two levels of need (high = +1, low = -1); 3) an additive term indicating high-merit partner trials (coded as 1/0); and 4) an additive term indicating low-merit partner trials (coded as 1/0). The *z*, *a*, and *ndt* parameters were fixed across need and merit conditions.
